# Long-Term High-Altitude Hypoxia and Alpha Adrenoceptor-Dependent Pulmonary Arterial Contractions in Fetal and Adult Sheep

**DOI:** 10.3389/fphys.2019.01032

**Published:** 2019-08-28

**Authors:** Dafne Moretta, Demosthenes G. Papamatheakis, Daniel P. Morris, Paresh C. Giri, Quintin Blood, Samuel Murray, Marian Ramzy, Monica Romero, Srilakshmi Vemulakonda, Sidney Lauw, Lawrence D. Longo, Lubo Zhang, Sean M. Wilson

**Affiliations:** ^1^Pulmonary and Critical Care, School of Medicine, Loma Linda University, Loma Linda, CA, United States; ^2^Pulmonary and Critical Care Medicine, UC San Diego Health, San Diego, CA, United States; ^3^Lawrence D. Longo MD Center for Perinatal Biology, School of Medicine, Loma Linda University, Loma Linda, CA, United States; ^4^Advanced Imaging and Microscopy Core, School of Medicine, Loma Linda University, Loma Linda, CA, United States

**Keywords:** pulmonary artery, hypoxia, calcium, contraction, adrenergic receptor, fetus, sheep

## Abstract

Autonomic innervation of the pulmonary vasculature triggers vasomotor contractility predominately through activation of alpha-adrenergic receptors (α-ARs) in the fetal circulation. Long-term hypoxia (LTH) modulates pulmonary vasoconstriction potentially through upregulation of α_1_-AR in the vasculature. Our study aimed to elucidate the role of α-AR in phenylephrine (PE)-induced pulmonary vascular contractility, comparing the effects of LTH in the fetal and adult periods on α-AR subtypes and PE-mediated Ca^2+^ responses and contractions. To address this, we performed wire myography, Ca^2+^ imaging, and mRNA analysis of pulmonary arteries from ewes and fetuses exposed to LTH or normoxia. Postnatal maturation depressed PE-mediated contractile responses. α_2_-AR activation contracted fetal vessels; however, this was suppressed by LTH. α_1A_- and α_1B_-AR subtypes contributed to arterial contractions in all groups. The α_1D_-AR was also important to contractility in fetal normoxic vessels and LTH mitigated its function. Postnatal maturity increased the number of myocytes with PE-triggered Ca^2+^ responses while LTH decreased the percentage of fetal myocytes reacting to PE. The difference between myocyte Ca^2+^ responsiveness and vessel contractility suggests that fetal arteries are sensitized to changes in Ca^2+^. The results illustrate that α-adrenergic signaling and vascular function change during development and that LTH modifies adrenergic signaling. These changes may represent components in the etiology of pulmonary vascular disease and foretell the therapeutic potential of adrenergic receptor antagonists in the treatment of pulmonary hypertension.

## Introduction

The pulmonary circulation is regulated to optimize respiratory gas exchange. Various factors affect pulmonary vascular reactivity, including neural, hormonal, inflammatory, and local mediators. Autonomic innervation of the pulmonary vasculature has been documented in various mammals although there is significant variation in the distribution of the nerves (Barnes and Liu, 1995). Considerable evidence also indicates there is a greater density of autonomic nerve fibers in larger vessels and vascular branching points (Daly and Hebb, 1966). The functional significance of sympathetic innervation of the human lung is not well understood although both α- and β-adrenergic receptors (ARs) are expressed in the pulmonary vascular bed. α-AR function predominates, however, in the fetal circulation with a higher basal vasomotor tone and greater reactivity to α-adrenergic stimulation (Mandel and Taichman, 2006). Notably, long-term hypoxia (LTH) results in upregulation of α_1_-AR gene transcription (Salvi, 1999).

Pulmonary vasoconstriction and high pulmonary vascular resistance, principally owing to a relatively low oxygen tension, are hallmarks of the fetal circulation. The high vascular resistance reduces energy expenditure to an organ that does not serve its primary purpose *in utero* (Weir et al., 2000). LTH during gestation due to high-altitude living, smoking, maternal anemia, placental insufficiency, or other causes is detrimental to the fetus and causes pulmonary hypertension and other complications in the newborn (Niermeyer, 2007). However, the underlying mechanisms remain largely elusive.

Previously, we demonstrated in sheep that LTH increased norepinephrine-induced contractions in pulmonary arteries and decreased acetylcholine-mediated relaxations in pulmonary veins (Xue et al., 2008). Other studies showed that phenylephrine (PE)-induced contractions were increased in endothelium intact but not denuded, pig pulmonary arteries exposed to hypoxia (Ogata et al., 1992). We also demonstrated that PE-induced cytosolic Ca^2+^ responses were similar in pulmonary arterial myocytes from fetal and adult sheep (Goyal et al., 2008). Nonetheless, little is known regarding the impact of LTH in the fetal and adult periods on adrenergic-mediated contractions or the underlying receptor-induced Ca^2+^ signals. The aim of the current studies was to determine the influence of long-term hypoxic stress on α-AR-dependent pulmonary arterial contractility. We tested the hypothesis that LTH in the fetal and adult periods enhance α-AR-dependent pulmonary arterial contractility. This was evaluated in intact arterial segments from fetal and adult sheep that lived at low altitude or were exposed to high-altitude hypoxia for 110+ days.

## Materials and Methods

### Experimental Animals

Animal handling was performed as per our previous studies over the past two decades including numerous studies on vessels of the pulmonary vasculature. Sheep were chosen for study because of their similar developmental profile to human infants, especially with regards to their lung development (Papamatheakis et al., 2013; Ducsay et al., 2018). Secondarily, the changes in lung structure and function are somewhat mild relative to other species and again similar to humans (Papamatheakis et al., 2013). Non-pregnant and pregnant ewes born at low altitudes were purchased from Nebeker Ranch (Lancaster, CA; 720 m) and transported to the Loma Linda University (353 m; arterial PaO_2_ = 95 ± 5 Torr) or were transported and acclimatized to high altitude (3,801 m, PaO_2_ = 60 ± 5 Torr) at the Barcroft Laboratory, White Mountain Research Station (Bishop, CA) for approximately 110 days (Kamitomo et al., 1993; Longo et al., 1996). Previous studies show that the PaO_2_ of fetal animals exposed to this level of hypoxia was roughly 20 Torr (Kamitomo et al., 1993; Papamatheakis et al., 2013). Animals acclimatized to high altitude were transported to the Loma Linda University, and shortly after arrival, a tracheal catheter was placed in the ewe, through which N_2_ flowed at a rate adjusted to maintain PaO_2_ at ~60 Torr (Kamitomo et al., 1994) until the time of the experimental study. Arterial blood gasses were monitored in the hypoxic animals several times each day and the N_2_ flow rate adjusted as needed to regulate maternal PaO_2_. Within 1–5 days after arriving at the university, anesthesia was induced with Ketamine (10 mg/kg IV) and Midazolam (5 mg/kg IV).The ewe was then placed in the supine position, intubated, and anesthesia maintained by inhalation of 1.5–2.5% Isoflurane in oxygen. Following tissue collection, sheep were sacrificed by intravenous injection of the proprietary euthanasia solution, Euthasol (2 ml/kg; Virbac, Ft. Worth, TX, USA). All tissue bath and calcium experimental procedures were performed as previously described (Goyal et al., 2011; Papamatheakis et al., 2011, 2012; Blum-Johnston et al., 2016; Shen et al., 2018) within the regulations of the Animal Welfare Act, the National Institutes of Health Guide for the Care and Use of Laboratory Animals, “The Guiding Principles in the Care and Use of Animals” approved by the Council of the American Physiological Society, and the Animal Care and Use Committee of the Loma Linda University. Although the animals were housed in a rarified environment at the White Mountain Research Station and then the low PaO_2_ was maintained at the Loma Linda University by breathing hypoxic gasses, all experimental studies were performed under normoxic conditions at the Loma Linda University. This differs from studies performed by researchers in Chile who use sheep housed at the Putre Research Station, International Center for Andean Studies at 3,600 m above sea level (Herrera et al., 2007, 2010). In comparison to work by researchers performed at Putre on sheep and other high-altitude research stations using various other animal models (Papamatheakis et al., 2013; Ducsay et al., 2018), our studies identify those changes and mechanisms that persist in low-altitude environments following high-altitude living and gestation. Our work using terrestrial high-altitude exposure also differs from studies performed on sheep exposed to hypoxia in chambers that have relatively short exposure periods (Allison et al., 2016; McGillick et al., 2017). When comparing the terrestrial and chamber models of exposure, the long durations of our exposures would restrict the number of sheep that could be examined each year. Tissues from a total of 106 experimental animals were examined for the studies of this report. This includes 22 adult normoxic (AN), 26 adult hypoxic (AH), 36 fetal normoxic (FN), and 25 fetal hypoxic (FH) animals. The long duration of exposure combined with the large numbers of animals used in our studies makes such chamber studies or performing studies at the White Mountain field station largely impractical. Even still, we are also fully aware that other phenomenon and mechanisms could be unmasked by performing studies at high-altitude research laboratories, such as the augmented hypoxic pulmonary vascular pressures we previously reported in 2-week-old newborn lambs born at high altitude (Blood et al., 2013).

### Tissue Preparation

Pulmonary arteries were dissected immediately from isolated lungs for contractility experiments from non-pregnant adult ewes or fetuses under normoxic conditions. Fourth and fifth order pulmonary arteries with internal diameters of about 500–700 μm were dissected free of parenchyma and cut into 5 mm long rings in ice-cold phosphate free balanced salt solution of the following composition (mM): 126 NaCl; 5 KCl; 10 HEPES; 1 MgCl_2_; 2 CaCl_2_; 10 glucose; pH 7.4 (adjusted with NaOH). To avoid complications from endothelium-mediated effects, the endothelium was disrupted by carefully rotating the artery on a small roughened hypodermic needle or on the mounting wire (Papamatheakis et al., 2011).

### Contraction Studies

**Figure 1:**
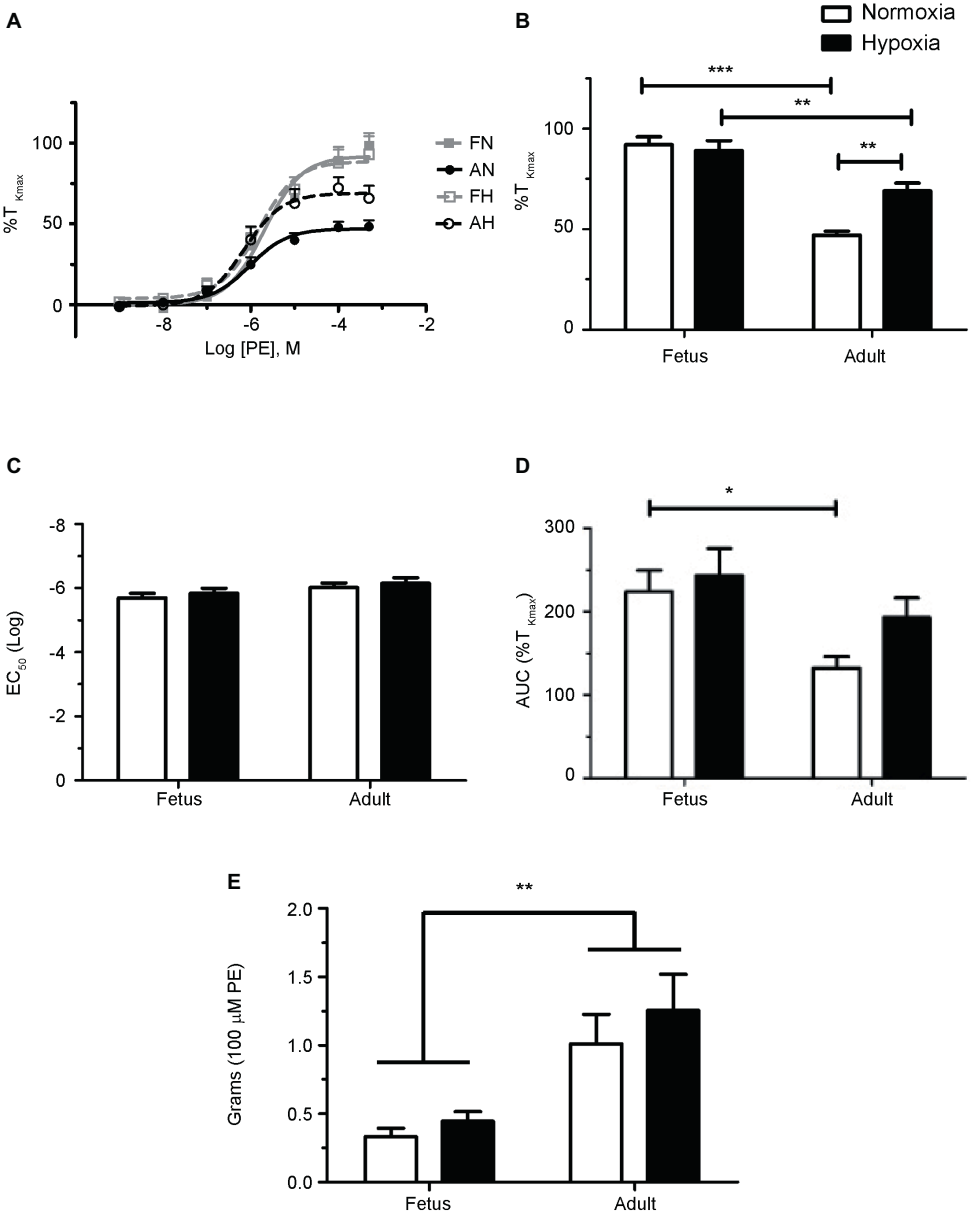
Phenylephrine-mediated contraction of pulmonary arteries from sheep is depressed in the adult period and enhanced by LTH. **(A)** Dose-response curves of pulmonary arterial rings exposed to 1 nM to 100 μM of phenylephrine in an additive manner from normoxic (solid lines) and LTH (dashed lines) fetal (gray lines and boxes) and adult (black lines and circles) sheep. Curves are plotted in relation to the maximal contraction induced by initial stimulation of 125 mM K^+^ Krebs-Henseleit solution (%T_Kmax_). **(B)** Maximum contraction relative to %T_Kmax_. **(C)** Log EC_50_ for phenylephrine induced contraction. **(D)** Area under the dose-response curve relative to high K^+^ contraction. **(E)** Absolute force developed for 100 μM phenylephrine-induced contraction. Points and bars are mean values and error bars indicate ± S.E.M. Comparisons between groups were made using a two-way ANOVA with a Bonferroni *post hoc* analysis (^*^p < 0.05, ^**^p < 0.01, ^***^p < 0.001). Numbers of animals for each group are provided in the “Methods” section.

**Figure 2:**
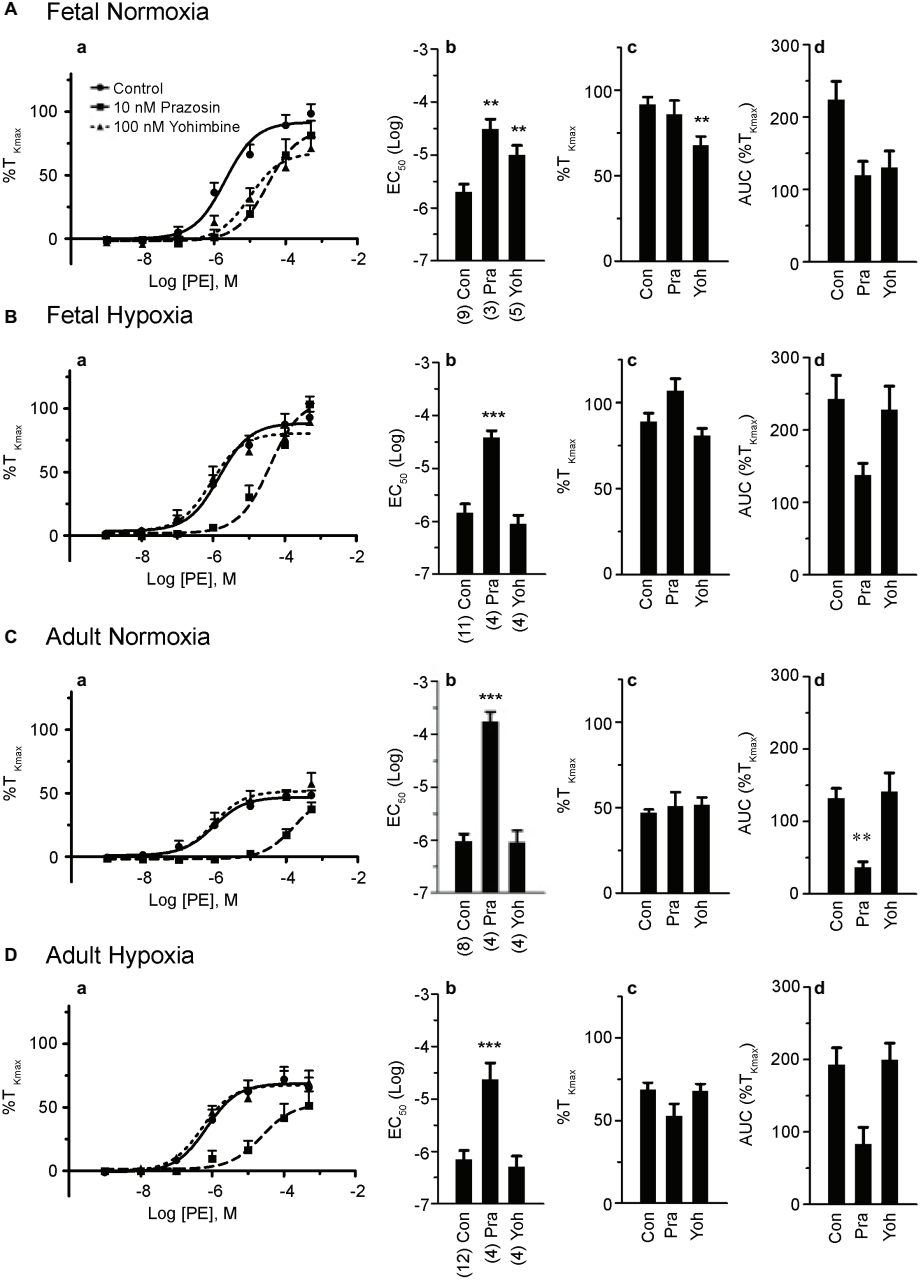
Phenylephrine-induced contraction is largely dependent on α_1_-adrenergic contraction. **(a)** Dose-response curves of pulmonary arterial rings exposed to 1 nM to 100 μM phenylephrine in an additive manner from normoxic and LTH, fetal **(A,B)** and adult **(C,D)** sheep. Solid lines with circles indicate vehicle-control (DMSO) while long dashes and squares indicate 10 nM prazosin and triangles and short dashes indicate 100 nM yohimbine. Log agonist vs. response curves are plotted in relation to the maximal contraction induced by initial stimulation of 125 mM K^+^ Krebs-Henseleit solution (%T_Kmax_). **(b)** Log EC_50_ for phenylephrine-induced contraction. **(c)** Maximum contraction relative to %T_Kmax_. **(d)** Area under the dose-response curve relative to high K^+^ contraction. Points and bars are mean values while error bars indicate ± S.E.M. Comparisons of drug treated arteries to control were made using a one-way ANOVA with a Newman-Keuls multiple comparison test (^**^p < 0.01, ^***^p < 0.001). Numbers of animals for each fetal and adult group are provided in the “Methods” section and in **(b)**.

**Figure 3:**
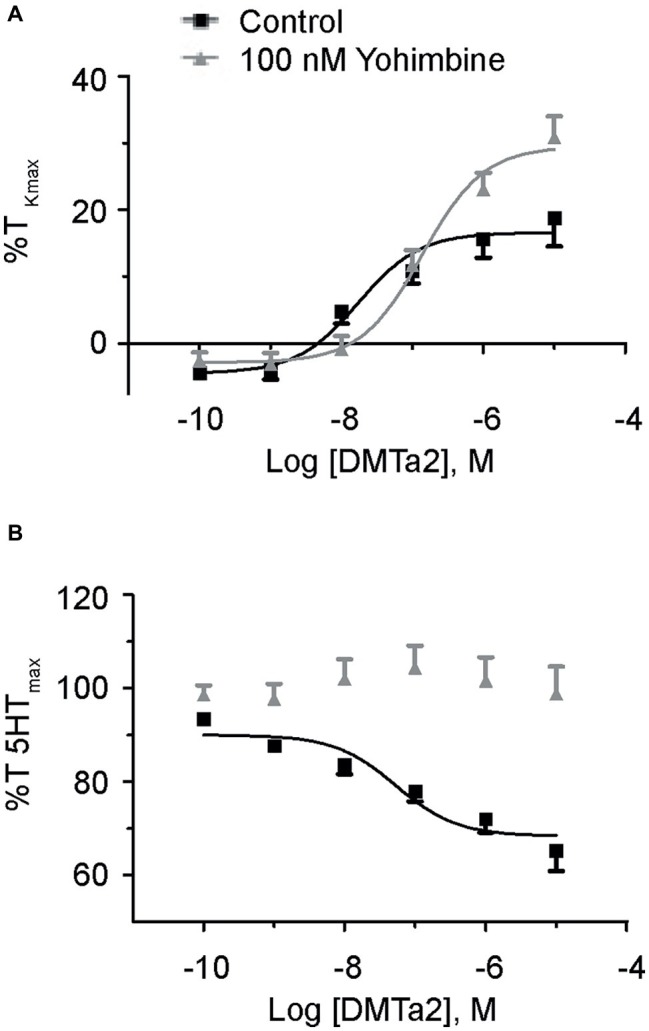
Dexmedetomidine causes contraction and relaxation of normoxic fetal pulmonary arteries. Dose-response curves of pulmonary arterial rings exposed to 100 pM to 10 μM of dexmedetomidine in an additive manner from normoxic fetal sheep in the absence **(A)** and presence **(B)** of 10 μM serotonin. Gray lines and triangles are for dexmedetomidine contraction **(A)** or relaxation **(B)** in the presence of yohimbine, and black lines and boxes are for dexmedetomidine contraction **(A)** or relaxation **(B)** in the presence of vehicle-control (DMSO). Solid lines indicate log agonist vs. response curves that are plotted in relation to the maximal contraction induced by initial stimulation of **(A)** 125 mM K^+^ Krebs-Henseleit solution (%T_Kmax_) or **(B)** 10 μM serotonin. Points and error bars indicate mean ± S.E.M. Numbers of animals for each group are provided in the “Methods” section.

**Figure 4:**
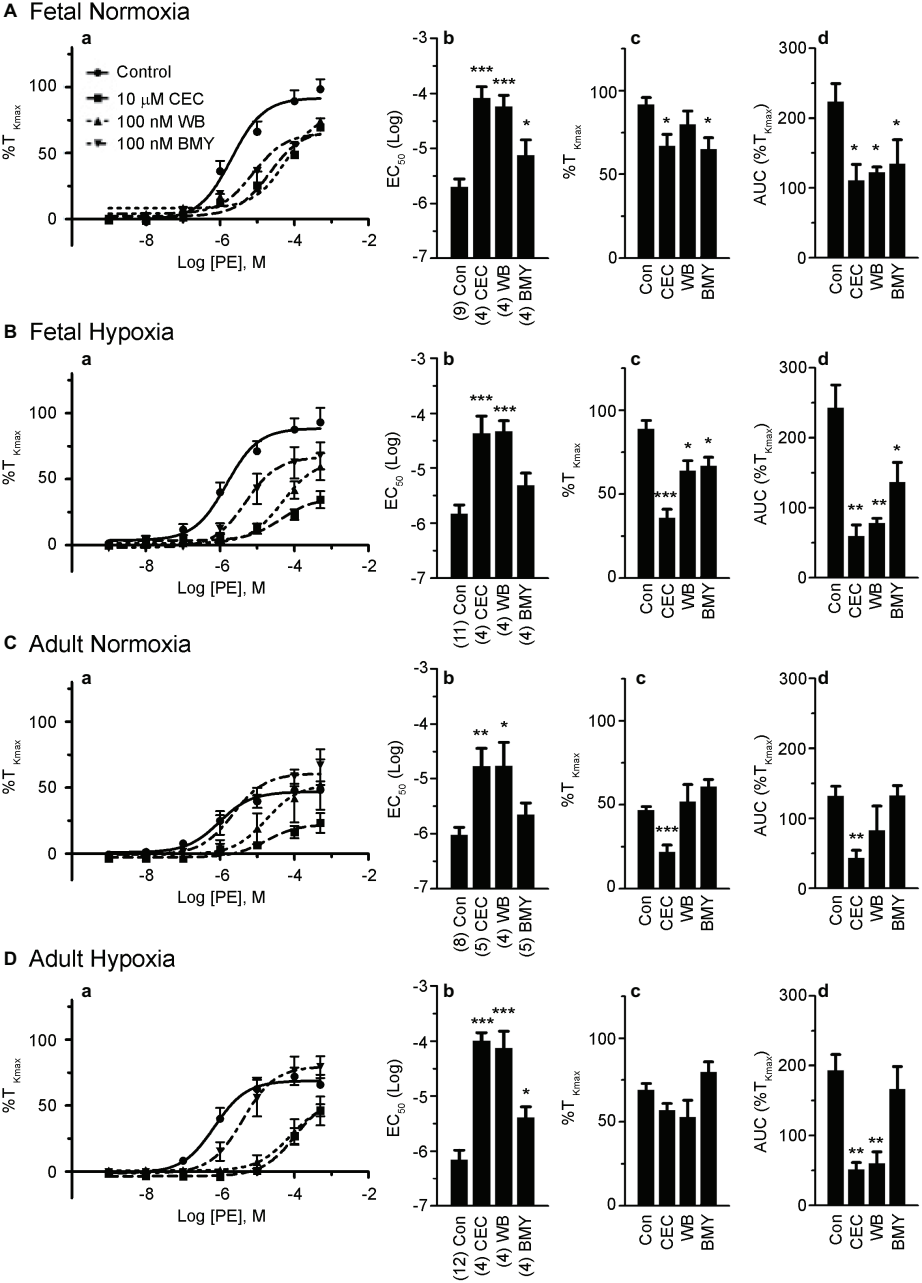
α_1A_-AR and α_1B_-AR antagonists preferentially block phenylephrine-induced pulmonary vascular contractility. **(a)** Dose response curves of pulmonary arterial rings exposed to 1 nM to 100 μM phenylephrine in an additive manner from normoxic and LTH, fetal **(A,B)** and adult **(C,D)** sheep. Curves of log agonist vs. response were repeated in the absence (circles and solid lines) or presence of 10 μM CEC (α_1B_-AR blocker, squares and long dashed lines), 100 nM WB (α_1A_-AR blocker, triangles and short dashed lines) or 100 nM BMY (α_1D_-AR blocker, upside down triangles and dashed and dotted lines) for normoxic and LTH, fetal **(A,B)** and adult **(C,D)** sheep pulmonary arteries. **(b)** Log EC_50_ for phenylephrine induced contraction. **(c)** Maximum contraction relative to high K^+^. **(d)** Area under the dose-response curve relative to high K^+^ contraction. Points and bars are mean values while error bars indicate ± S.E.M. Comparisons of drug treated arteries to control were made using a one-way ANOVA with a Newman-Keuls Multiple Comparison Test (^*^p < 0.05, ^**^p < 0.01, ^***^p < 0.001). Numbers of animals for each fetal and adult group are provided in the “Methods” section and **(b)**.

**Figure 5:**
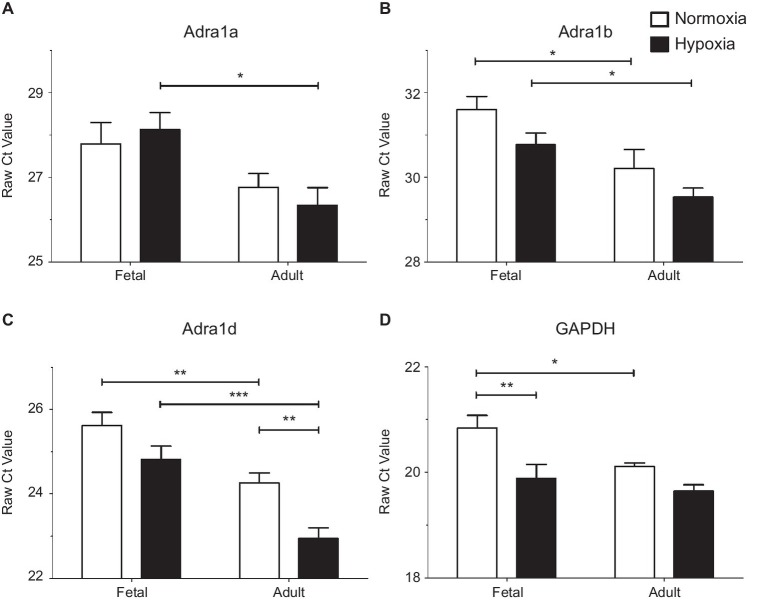
LTH in the fetal and adult periods have unique impact on mRNA expression of alpha adrenergic receptors. Presented are the raw Cycle threshold (Ct) values from real time quantitative PCR measurements for **(A)** Alpha 1a AR, **(B)** Alpha 1b AR, **(C)** Alpha 1d AR, and **(D)** GAPDH. Normalization was to input RNA, all PCR reactions used input cDNA derived from 5 ng of total RNA. Bars and error bars represent the mean ± S.E.M. Significant differences between groups were established using a two-way ANOVA with a Bonferroni *post hoc* analysis (^*^p < 0.05, ^**^p < 0.01, ^***^p < 0.001). Numbers of animals for each group are provided in the methods section.

**Figure 6:**
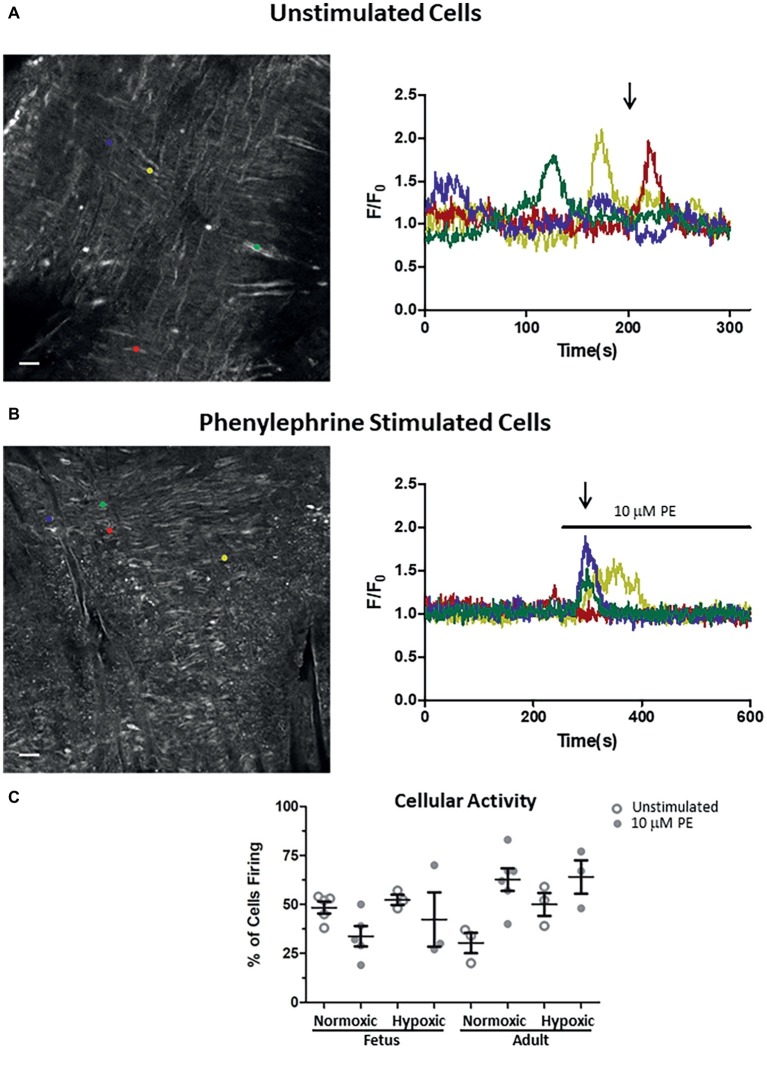
Impact of LTH in the fetal and adult periods on unstimulated and PE-mediated whole-cell Ca^2+^ responses in pulmonary arterial myocytes. Shown are representative fluorescent images at time points shown by the arrow and the traces of fractional Fluo-4 fluorescence for unstimulated **(A)** and 10 μM phenylephrine stimulated **(B)** arteries. **(C)** Percentage of myocytes in a 1,000 μm^2^ area that had Ca^2+^ responses to 10 μM PE under unstimulated (open circles) and stimulated (filled circles) conditions based on the number of runs examined. Colored circles on each image correspond to the placement of regions of interest for the traces of the same color in the tracing. About 10 μM phenylephrine was present during the time period denoted by the horizontal bar. Images were made with a 20× non-immersion objective (NA 0.8). Scale bar is 25 μm. Numbers of animals for each group are provided in the “Methods” section.

Pulmonary arterial contraction studies were performed as previously described at the Loma Linda University at 353 m (Blum-Johnston et al., 2016; Giang et al., 2016). In brief, pulmonary arterial rings on tungsten wires were suspended in organ baths (Radnoti Glass Instruments, Inc. Monrovia, CA) that contained 5 or 10 ml of modified Krebs-Henseleit solution containing in mM: 120 NaCl; 4.8 KCl; 1.2 K_2_HPO_4_; 25 NaHCO_3_; 1.2 MgCl_2_; 2.5 CaCl_2_; 10 glucose. The bath solution was maintained at 37°C and aerated with 95% O_2_ to 5% CO_2_ (pH = 7.4). The wires were attached to low compliance force transducers (Radnoti Glass Instruments Inc.) for the measurement of isometric force (Blum-Johnston et al., 2016; Giang et al., 2016) that were connected to an analogue to digital data interface (Powerlab 16/30 A/D Instruments, Colorado Springs, CO; or MP100, Biopac, Goleta, CA) attached to a computer. The changes in tension were recorded using Chart 5.5 (AD Instruments, Colorado Springs, CO) or AcqKnowledge 3.9 (Biopac, Systems, Inc., Goleta, CA) and analyzed *post hoc*. Vessels were equilibrated without tension for a minimum of 30 min and tensioned to approximately 0.75 g (Goyal et al., 2011; Papamatheakis et al., 2011; Blum-Johnston et al., 2016). Arterial tension was normalized to a maximum response obtained with 125 mM KCl (high K^+^) (%T_Kmax_) or 10 μM serotonin (%T_5HTmax_). For evaluating dose-response characteristics, arteries were stimulated by applying 1 nM to 100 μM phenylephrine or 100 pM to 10 μM dexmedetomidine (DMT) logarithmically without washing in-between each concentration increase. Figure 1 studies were based on arterial segments isolated from normoxic fetal (9), adult (8) and hypoxic fetal (11), and adult (12) animals, which served as the controls for the pharmacological studies outlined in Figures 2, 4. Figure 2 studies were based on arteries isolated from normoxic fetal prazosine (3) and yohimbine (5); adult prazosine (4) and yohimbine (4); as well as hypoxic fetal prazosine (4) and yohimbine (4); adult prazosine (4) and yohimbine (4). Figure 3 studies were based on arteries isolated from fetal normoxic animals for control (3) and yohimbine (4) studies. Figure 4 studies were based on arteries isolated from normoxic fetal CEC (4), WB (4), and BMY (4); adult CEC (5), WB (4), and BMY (5); as well as hypoxic fetal CEC (4), WB (4), and BMY (4); adult CEC (4), WB (4), and BMY (4). Figure 5 measurements were made in six animals from each group except for the fetal normoxic group, which was based on tissues from seven animals. Figure 6 recordings were made in three adult normoxic, three adult hypoxic, five fetal normoxic, and three fetal hypoxic animals.

### RNA Purification

To obtain RNA of adequate quality for qPCR analysis from fibrous sheep arteries, a protocol combining the Qiagen® Fibrous Tissue RNeasy kit (Qiagen, Germantown, MD) with a Bullet blender (Next Advance Inc.) was developed for mid-sized pulmonary arteries as described below. Notably, the α-AR subtypes in native tissue were expected to have low expression (Michel et al., 2009; Schonbrunn, 2014), increasing concerns about the accuracy of the qPCR measurements. A 450 μl aliquot of buffer RLT with Mercaptoethanol was placed into the RNAse-free Blue Rhino Bead beating tube at room temperature. RNA later treated vessels that were frozen in liquid N_2_ and stored at −80°C were then placed into the tube and the bullet blender unit was run for 3 min at maximum setting. Subsequently, 885 μl RNase-free H_2_O and 15 μl of protease K were added, and samples were then mixed and incubated at 55°C in a water bath for 10–11 min. Sample tubes were then spun at 10,000 × *g* for 3 min at room temperature and the supernatant transferred to RNase free tubes followed by addition of 0.5 vol. of Mol. Bio grade absolute EtOH. The samples were loaded onto RNeasy columns and the fibrous tissue protocol completed with on-column DNAse treatment and elution using two 30 μl additions of RNAse-free H_2_O. RNA concentrations were quantified by Nanodrop and RNA integrity demonstrated on bleach agarose gels (Aranda et al., 2012). For all samples, the large RNA subunit was clearly predominant relative to the smaller subunit, presenting the appearance of RNA previously demonstrated to have RIN values between 7 and 8. The final RNA samples were aliquoted, frozen in liquid N_2_, and stored at −80°C.

### Reverse Transcription

The QuantiTect Reverse Transcription Kit (Qiagen), which incorporates a second DNAse treatment, was used according to the manufacturer recommended protocol. This included 400 ng input RNA for each 10 μl RT reaction. All reactions were set up on ice to minimize the risk of RNA degradation. Control experiments without reverse transcriptase confirmed that the cDNA samples did not have any genomic sequence contamination as judged by the absence of target product in trans-intron qPCR reactions.

Analysis of mRNA levels was based on the CFX96 Real-Time PCR Detector and software (Biorad, Hercules CA). All qPCR reactions used cDNA derived from 5 ng of reverse transcribed total RNA. Multiple primer pairs were tested to establish the most reliable primers for each receptor subtype based on PCR efficiency, linearity within the analytic range, and localization within essential coding sequence in the second exon of common subtype isoforms. The best primer pair for each subtype positioned within the essential coding sequence in the second exon of common isoforms (Hawrylyshyn et al., 2004) was used for qPCR quantitation. These primers included those targeted to: adra1a Forward, ATACCCATGCTCCAGTCAAG; Reverse, GTGTTTGGAGGACTGCTTTC. adra1b Forward, TGTTCAAGGTGGTCTTCTGG; Reverse, GCTTGAACTCCTTGCTGGA. adra1d Forward, AGGGCGTCTTCAAGGTTATC; Reverse, CAGGGGTAGATGAGTGGATT. GAPDH, Forward, TGGAAATGTATGGAGGTCGG; Reverse, GGAAGAGAGAGTTCCTCAGC.

Initial experiments included both β-actin and GAPDH as potential internal load corrections; however, all applications of the ∆∆Ct approach produced increased variance consequently, normalization was to input cDNA (derived from 5 ng total RNA), and statistical analysis was based on raw, average Ct values produced by three qPCR quantifications on separate days. For each experiment, singly frozen sub-aliquots of sheep genomic DNA (Amsbio LLC) were used as a reference to estimate transcript copies per cell for each of the three α_1_-AR subtypes. Quantitative estimates of copy number were based on ∆Ct from 316 double strand copies assuming one cycle represented a two-fold change in copy number. Estimates of mRNA copies per cell are based on the assumption of 20 pg total RNA per cell, which implies 5 ng input total RNA representing ~250 cells.

### Calcium Imaging

Cytosolic calcium was measured in pulmonary arterial myocytes *in situ* using an *en face* preparation in balanced salt solution at the Loma Linda University, as we have recently described (Goyal et al., 2011; Papamatheakis et al., 2011; Blum-Johnston et al., 2016). In brief, tissues were loaded with the Ca^2+^ sensitive dye Fluo-4 AM (10 μM, Invitrogen, Carlsbad, CA, USA) with 0.1% pluronic F127 for 1.5–2 h at room temperature in the dark and measurements made with a Zeiss 710 NLO laser scanning confocal imaging workstation (Thornwood, NY) mounted to an inverted microscope (Zeiss AxioObserver A1). Arterial segments were then washed for 30 min, cut into linear strips, pinned to sylgard blocks (Ellsworth Adhesives, Germantown, WI), placed in an open bath imaging chamber (Warner Instruments, Hamden, CT), and mounted *en face* on the confocal imaging stage. Arteries were perfused at approximately 1 ml/min using a peristaltic pump (Rainin, Oakland, CA) with an electronic pinch valve system (Automate Scientific, Berkeley, CA). Cells were illuminated at 488 nm with a krypton argon laser, and the emitted light was collected using a photomultiplier tube (frames of 512 × 512 pixels) and a prismatic defractor (wavelengths of 493–622 nm). Full frame images were generated every 1 s. The pinhole was adjusted to provide an imaging depth of ~10 μm, which is roughly equivalent to the width of two cells (Goyal et al., 2011; Papamatheakis et al., 2011). Images were acquired at a 16-bit sampling depth using a non-immersion 20× Plan Apochromat (numerical aperture, NA 0.8). Regions of interest were examined *post hoc*, and although attempts were made to analyze, the oscillations in fluorescent intensity from recordings using LCPro quantification could not be adequately performed due to significant vasomotion during PE stimulation (Francis et al., 2012, 2014). Because of the substantial vessel reactivity, the numbers of arterial myocytes with Ca^2+^ events due to phenylephrine were recorded and the percentage of active myocytes calculated by comparing to the numbers of cells with observable Flou-4 fluorescence in the region of interest (Goyal et al., 2011; Papamatheakis et al., 2011). The number of observable cells examined for Figure 6C in each 1,000 μm^2^ region were as follows: Adult Normoxic, AN, 6.6 ± 0.3; Adult Hypoxic, AH, 8.3 ± 0.6; Fetal Normoxic, FN, 7.6 ± 0.4; Fetal Hypoxic, FH, 7.0 ± 0.2.

### Chemicals and Drugs

Most reagents and chemicals were purchased from Sigma-Aldrich (St. Louis, MO) except dexmedetomidine which was purchased from Tocris and other chemicals and reagents as noted.

### Statistical Methods

All time-series recordings were graphed, and statistical analyses were made using GraphPad Prism 5.0 (La Jolla, CA) with the data presented as mean ± S.E.M. Data for bar plots were examined for normality with a D’Agostino and Pearson omnibus normality test. For most contractility, confocal, and PCR studies, comparisons were made within and among groups using either a two-way ANOVA and Bonferroni *post hoc* analyses or a one-way ANOVA with a with a Newman-Keuls Multiple Comparison Test. Categorical data shown in Figure 6C were analyzed by a chi-square test. Dose-response curves were fitted in Prism 5.0 using a Hill equation (Goyal et al., 2011; Papamatheakis et al., 2011; Blum-Johnston et al., 2016). Comparisons of the non-linear fit data for the influence of yohimbine on serotonin contraction, Figure 3A were examined by an extra sum of squares F-test. The *N* values reported reflect the total number of regions of interest, arterial segments, and/or total number of sheep tested as delineated elsewhere in the methods, figure legends, and Table 1. *p* < 0.05 was accepted as statistically significant.

**Table 1:** Chronic hypoxia, maturation and adrenergic receptor and GAPDH mRNA expression.

Group	Mean ± SEM	*N*	Fold change	mRNA copies per cell
Adra1a				
FN	27.79 ± 0.51	7	1.00	0.40
FH	28.13 ± 0.40	6	0.79	0.32
AN	26.77 ± 0.32	6	2.03	0.81
AH	26.35 ± 0.41a	6	2.73	1.09
Adra1b				
FN	31.60 ± 0.31	7	1.00	0.63
FH	30.77 ± 0.28	6	1.78	1.12
AN	30.50 ± 0.45a	6	2.15	1.35
AH	29.54 ± 0.21a	6	4.19	2.65
Adra1d				
FN	25.62 ± 0.32	7	1.00	2.29
FH	24.81 ± 0.32	6	1.75	4.00
AN	24.26 ± 0.23a	6	2.56	5.86
AH	22.95 ± 0.25a,b	6	6.35	14.54
GAPDH				
FN	20.84 ± 0.24	7	1.00	20.1
FH	19.89 ± 0.27b	6	1.94	39.1
AN	20.11 ± 0.07a	6	1.66	33.4
AH	19.65 ± 0.12	6	2.29	46.1

*qPCR data presented as raw Ct values produced by 5 ng input RNA as shown graphically in Figure 5. N indicates the number of animals for each condition in each group. Fold change is calculated relative to FN condition for each group. mRNA copies/cell estimated as described in methods. Significance established using two-way ANOVA with Bonferroni post hoc analysis. a – significant relative to normoxic fetus, b – significant compared to normoxic counterpart. Actual p’s are provided in Figure 5*.

## Results

### Maturation and Long-Term Hypoxia Influence Alpha-Adrenergic-Dependent Reactivity

The first series of experiments were designed to compare the effects of LTH in the fetal and adult periods on sympathetic-induced contractions of pulmonary arteries. This was achieved by examining the influence of cumulative doses of the selective α-AR agonist phenylephrine on contractions of pulmonary vascular rings isolated from adult and fetal sheep housed in either normoxic or LTH conditions. Phenylephrine was chosen *in lieu* of norepinephrine because the latter activates β-ARs that are expressed in pulmonary arteries (Hyman and Kadowitz, 1989; Magnenant et al., 2003). Figure 1 depicts summarized data for dose-response curves that were produced from these experiments. For all groups, arterial contractions in response to PE displayed a sigmoidal dose-response relationship (Figure 1A) and similar EC_50_’s (Figure 1C), indicating that neither development nor LTH modified the potency of PE. However, the maximum contraction relative to high potassium in the adult period for normoxic sheep was significantly reduced relative to fetus as shown in Figure 1B. While LTH had no effect on fetal pulmonary arterial contractions, it elevated the maximum contraction in the adult period to near fetal levels as illustrated in Figure 1B and in the area under the curve as shown in Figure 1D. Although maximal contraction relative to T_Kmax_ was lower in adult compared to fetus (Figure 1B), the absolute contractile force due to phenylephrine was higher in the adult (Figure 1E).

### Maturation and Long-Term Hypoxia Impact the Role of α_1-_ Versus α_2_-Adrenergic Receptors to Arterial Contraction

Based on these results and the literature to date (Levy et al., 1995; MacLean and McCulloch, 1998; Magnenant et al., 2003; Oriowo et al., 2003; Gornemann et al., 2007), we performed a series of experiments in order to determine the contributions of α_1_- and α_2_-AR to pulmonary arterial contractions. This was achieved by repeating the dose-response curves for each of the four animal groups in the presence or absence of antagonists of α_1_-AR (10 nM prazosin) or α_2_-AR (100 nM yohimbine) (Schindler et al., 2004; Gornemann et al., 2009). Data for these studies are shown in Figure 2. Prazosin had a similar effect in all four groups (Figure 2), causing a substantial rightward shift in the dose-response relationships (Figures 2Aa–Da) that translated into a reduced potency for PE in all groups (Figures 2Ab–Db). In comparison, prazosin had little or no effect on efficacy (Figures 2Ac–Dc). Figures 2Ad–Dd show that the area under the contractile curve was not as sensitive to prazosin or yohimbine as compared to the potency. Yohimbine caused a significant rightward shift in the PE-mediated contractility curve only in vessels from the fetal normoxic group (Figure 1Ac) but had no effect on PE-mediated contractions of adult vessels (normoxic and hypoxic) or fetal hypoxic vessels. These results suggest that α_2_-ARs may contribute to sympathetic contraction before birth and that antenatal hypoxia and the adult period suppress this role.

### α_2_-Adrenergic Receptors Are Important to Contractile and Relaxation Responses in Fetal Normoxic Pulmonary Arteries

Because yohimbine blocked PE-mediated contractions only in arteries from the normoxic fetal group, studies were performed to further delineate the potential influence of α_2_-AR activation on arterial contraction (Shebuski et al., 1987; Jantschak and Pertz, 2012) as well as relaxation (Magnenant et al., 2003) in the fetal normoxic group. Evaluation of vessel reactivity was achieved by stimulating normoxic fetal arteries with the selective α_2_-AR agonist, dexmedetomidine (Toyama et al., 2009). Figure 3A shows the dose-response curves for normoxic fetal arteries stimulated with increasing doses of dexmedetomidine in the absence or presence of yohimbine. Dexmedetomidine caused modest contractions with a maximal response of approximately one-fifth that induced by PE (Figure 2A). Yohimbine treatment shifted the dose-response curve for dexmedetomidine contraction by one-log to the right (−7.8 ± 0.25 vs. −6.9 ± 0.14, *p* < 0.05). This confirms that α_2_-ARs are involved in vasomotor tone in the fetal pulmonary circulation, and this effect can be mitigated by α_2_-AR antagonists like yohimbine.

We then sought to evaluate the role of α_2_-AR to cause further contraction as occurs in pre-stimulated pulmonary vasculature of dog (Shebuski et al., 1987) and rat aorta (Ok et al., 2016), or whether the arteries will relax as described in pig pulmonary arteries (Pepke-Zaba et al., 1993) and fetal sheep pulmonary vasculature (Magnenant et al., 2003). Figure 3B shows the dose-response curves of fetal normoxic arteries treated with incremental doses of dexmedetomidine that were pre-stimulated with 10 μM serotonin. Our results illustrate that serotonin precontracted arteries relaxed in response to dexmedetomidine, an effect which was blocked by yohimbine. Taken together, these results indicate that inhibition of α_2_-AR does not seem to have a significant impact on phenylephrine-induced contraction in vessels from hypoxic animals from the fetal or adult periods but preferentially attenuates contractions in fetal normoxic vessels. However, activation of α_2_-AR in fetal normoxic vessels can cause vessel relaxation when arteries have significant serotonergic pretone.

### α_1A_-, α_1B_-, and α_1D_-Adrenergic Receptors Have Selective Roles to Vessel Contraction

The roles of the different α_1_-AR subtypes to the contraction responses shown in Figures 1, 2 were further delineated by performing PE dose-response contraction studies in the presence or absence of subtype selective α_1_-AR antagonists. The antagonists were chosen based on previously shown selectivity for the three α_1_-AR subtypes, namely α_1A_-AR (100 nM WB 4101), α_1B_-AR (10 μM CEC), and α_1D_-AR (100 nM BMY 7378) (Goyal et al., 2010b, 2014). The results of these experiments are illustrated in Figure 4. Vessels from the fetal normoxic period were affected by the selective inhibitors of the three α_1_ subtypes, where the dose-response curves (Figure 4Aa) and corresponding EC_50_ (Figure 4Ab) were significantly rightshifted and the areas under the curve were decreased (Figure 4Ad). The efficacy of contraction (Figure 4Ac) was less sensitive than the other parameters, being reduced by inhibitors of α_1B_-AR and α_1D_-AR but not α_1A_-AR. Similarly, Figure 4B shows the influence of inhibitors on vessels from the fetal hypoxic period. All three inhibitors reduced the amplitude of contraction and area under the curve (Figures 4Ba,Bc,Bd). The potency of contraction, however, was rightshifted by inhibitors of α_1A_-AR and α_1B_-AR but not α_1D_-AR (Figure 4Bb).

Vessels from the adult normoxic period were sensitive to α_1A_-AR and α_1B_-AR but much less dependent on the α_1D_-AR. The EC_50_ was rightshifted by inhibitors of both α_1A_-AR and α_1B_-AR (Figures 4Ca,Cb) while efficacy of contraction and area under the curve were each reduced by inhibition of α_1B_-AR (Figures 4Cc,Cd). In comparison, contractility of arteries from the adult hypoxic period was affected by inhibitors of all three α_1_-AR receptors, but the effect for the α_1D_-AR was marginal. Although the amplitude was insensitive to the inhibitors (Figure 4Dc), the potency was rightshifted substantially by inhibitors of α_1A_-AR and α_1B_-AR and to a lesser extent by α_1D_-AR (Figures 4Da,Dc). The area under the curve was reduced by inhibitors of α_1A_-AR and α_1B_-AR but not α_1D_-AR (Figure 4Dd).

### mRNA Expression of α_1A_-, α_1B_-, and α_1D_-Adrenergic Receptor

Subsequently, qPCR was used to investigate subtype expression to determine if these patterns account for the changes observed in our functional studies of pulmonary arteries (Figure 5 and Table 1). Analysis of α_1A_-AR expression showed that message levels of this adrenergic receptor subtype did not increase with hypoxia in fetal or adult vessels but did increase in the adult period relative to fetus as shown in Figure 5A by decreased Ct values and by the increase in fold change and mRNA copies per cell (Table 1). Statistical analysis was performed on raw Ct values because during assay development, attempts to apply the ∆∆Ct method using actin and/or GAPDH as internal controls were ineffective, as these corrections substantially increased variance. Further, GAPDH expression increased under hypoxic conditions in the fetus (Figure 5D and Table 1), much as occurs in bovine endothelial cells (Graven et al., 1994; Graven and Farber, 1995). Of note, the tight clustering of GAPDH measurements for the normoxic adult suggest the variability observed in the other sample groupings reflects real differences in subunit expression. As for the α_1A_-AR, the α_1B_-AR and α_1D_-AR increased expression in the adult period relative to the fetus (Figures 5B,C and Table 1). The only significant difference due to hypoxia was an approximately 2.5-fold increase observed for α_1D_-AR in adult vessels.

### Alpha-Adrenergic Receptor Generated Ca^2+^ Responses

The final series of studies examined the ability of PE to cause Ca^2+^ responses in myocytes that line the arterial wall. These studies were performed using an *en face* preparation with confocal laser scanning microscopy approaches such as we have used previously to examine serotonergic as well as caffeine-associated stimulation of Ca^2+^ responses in pulmonary arterial myocytes (Goyal et al., 2011; Papamatheakis et al., 2011; Hadley et al., 2012). In the present study, cytosolic Ca^2+^ of the pulmonary arterial myocytes was monitored under unstimulated conditions and in response to addition of 10 μM PE. These studies were performed based on our prior experiments where PE caused cytosolic Ca^2+^ elevations in isolated myocytes from pulmonary arteries of fetal and adult sheep (Goyal et al., 2008). Moreover, these Ca^2+^ responses are likely coupled to pulmonary arterial contractions (Goyal et al., 2010b, 2011, 2014; Papamatheakis et al., 2011). The Ca^2+^-signaling behavior of the regions of interest are expressed as the average baseline-subtracted fractional fluorescence intensity tracing (F/Fo) over time and shown in the time series graphs in Figures 6A,B in the presence of extracellular Ca^2+^. The bar above the tracing in Figure 6B shows when 10 μM PE was applied to the tissue. The images in Figures 6A,B show *in situ* fluorescence micrographs of myocytes in the arterial wall isolated from normoxic fetal sheep for the time points as shown by the arrows in unstimulated (Figure 6A) and phenylephrine-stimulated (Figure 6B) conditions for the fluorescence intensity tracings in the panels shown on the right. Regions of interest are denoted by the small colored circles in the image panels and represent subcellular regions within individual myocytes that correspond to associated fluorescent time series data traces of the same color in Figures 6A,B. Figure 6A illustrates that under unstimulated conditions, the Ca^2+^ oscillations are stochastic, occurring randomly during the recording. Figure 6B shows that stimulation with 10 μM PE causes deterministic Ca^2+^ responses among the arterial myocytes. Figure 6C shows the number of responsive cells in the arterial wall for the data recordings as determined by examining the number of myocytes with calcium responses in 33 μm × 33 μm boxes (1,000 μm^2^). The figure shows that there is some variability in the percentage of myocytes with Ca^2+^ oscillations under control conditions and in response to phenylephrine stimulation, though there are no significant differences due to stimulation or in the fetal or adult periods.

## Discussion

### Summary of Findings

The present study provides new information systematically comparing the effects of LTH in the fetal and adult periods on the functional changes for the role of α_1_- and α_2_-adrenergic receptors in the pulmonary vasculature. The contractile response to phenylephrine underwent significant changes from the fetal to adult periods and LTH caused further alterations. Of note, α_2_-ARs appeared to be important to vascular reactivity before birth in the normoxic fetal period; however, these α_2_-AR effects were not present in the adult period and were lost in fetuses exposed to LTH. We found there were significant roles for α_1A_- and α_1B_-ARs in pulmonary arterial contractions of all groups examined. There was also a lesser role for α_1D_-AR in the fetus that was reduced by hypoxia and largely absent in the adult period. Developmentally, the contractile properties produced by adrenergic stimulation are complex and take place in the context of changing vessel behavior due to LTH exposure.

### Development and Alpha-Adrenergic Receptor Pulmonary Arterial Contractility

Developmental changes in adrenergic signaling of pulmonary arteries were expected given the dramatically different role these arteries play during the fetal period where blood flow is restricted as compared to the adult period where robust blood flow is required to exchange gasses. Given the need to maintain oxygenation in stressful situations, it is not surprising that adrenergic contractions during the adult period lessen relative to contractions due to depolarization with high K^+^. A similar reduction in adrenergic mediated contractions in the adult period occurs in the piglet, which has reduced norepinephrine mediated pulmonary vascular contractions following birth (Schindler et al., 2004). In this regard, our data showed that pulmonary vessels in adult sheep lost the contractile effects from both α_2_-AR activity and coupling to the α_1D_-AR, which were observed in the normoxic fetal period. Nevertheless, this relative decrease in contractility must be viewed in context as the absolute contractile force due to PE increased following birth. It is reasonable to suggest that the increased absolute force caused by PE is partly mediated through an increased expression of the α_1A_- and α_1B_-ARs. While it appears contradictory for the α_1D_-AR to play a minimal role in contractions of adult vessels given the 2.5-fold increases in its mRNA expression, α_1D_-AR levels do not necessarily correlate with arterial reactivity in arteries and instead contribute significantly to vascular remodeling in hypoxia-related pulmonary hypertension (Faber et al., 2007).

Because autonomic innervation of the lung likely increases after birth, our data suggest that postnatal maturation may result in other functional changes (Garcia-Sainz et al., 2001; Thiriet, 2013; Castillo-Badillo et al., 2015). Indeed, the process by which maturity into adulthood affects adrenergic dependent vascular contractility may be complex. One possibility is that during the adult period the increased sympathetic nerve stimulation may cause α adrenergic receptor desensitization and downregulation (Garcia-Sainz et al., 2001; Castillo-Badillo et al., 2015). Another explanation for decrements relative to high K^+^ during the adult period is that the sympathetic nerve supply may be trimmed. This tenant is supported by the lack of a profound difference in cellular Ca^2+^ signaling activity in fetal relative to adult pulmonary arterial myocytes.

### Long-Term Hypoxia and Alpha-Adrenergic Receptor Pulmonary Arterial Contractility

The LTH-induced enhancement of phenylephrine reactivity in pulmonary arteries of the adult period was expected. Chronic hypoxia in piglets prevents postbirth decreases in adrenergic tone (Schindler et al., 2004) and has been linked to hypoxia-induced pulmonary hypertension in multiple animal species including sheep (Llanos et al., 2011). This tie between adrenergic signaling and hypoxia-related pulmonary hypertension may be due to various factors. Hypertrophy of the smooth muscle layer in the small pulmonary arteries and arterioles appears to be a major determinant of pulmonary vascular responses to hypoxia in humans and other species (Tucker et al., 1975). Potentially, the thickness of the smooth muscle layer may be linked to the adrenergic receptor density and strength of stimulation (Xiao et al., 2003) and LTH in the fetal and adult periods may enhance adrenergic-mediated cellular hypertrophy and proliferation as occurs in cultured rat pulmonary arteries (Faber et al., 2006). The influence of LTH on adrenergic stimulation may be similar to chronic intermittent hypoxia, which increases sympathetic nervous system outflow from the brainstem (Zoccal et al., 2008). Neurohumoral modulatory pathways influenced by sympathetic stimulation include downregulation of nitric oxide (NO) signaling in the brain (Patel et al., 2001) and upregulation of endothelin-1 (Huang et al., 2010). Furthermore, physiological as well as psychological and environmental stresses are known to increase sympathetic nerve activity (Anderson et al., 1991). Chronic intermittent hypoxia also elicits chemosensory plasticity, including increased basal discharge, enhanced hypoxic sensitivity, and sensory long-term facilitation (Peng et al., 2003). Even still, the effect of LTH in the fetal and adult periods in sheep is distinct from the adaptive responses in chick embryos. Chronic hypoxia in 19-day-old chick embryos reduces pulmonary arterial contractions due to high K^+^, norepinephrine, U46119, and endothelin-1, although there is no difference in the perivascular innervation density before birth and contraction deficits resolve after birth (Villamor et al., 2004). These distinctions in prenatal programming effects between fetal sheep versus chick embryos are not unfounded and follow from studies by us and others in the cardiovascular systems of these models (Crossley et al., 2003; Villamor et al., 2004; Goyal et al., 2011; Blum-Johnston et al., 2016; Giang et al., 2016).

Adrenergic contraction of fetal pulmonary vessels involves all three α_1_-AR subtypes and was efficiently coupled to the contraction as PE stimulation produced about the same force as high K^+^. The role for α_2_-adrenoreceptors in the fetus was unanticipated as PE is fairly selective for the α_1_-AR; however, α_2_-ARs appear to be important for sympathetic pulmonary arterial contraction before birth and antenatal LTH attenuates their role in fetal vessels. Prior studies indicated that α_2_-ARs were expressed in the pulmonary vasculature and involved in arterial contractions, even before birth (Magnenant et al., 2003; Jantschak and Pertz, 2012). Much like our studies, postnatal maturation seems to contribute to downregulation of α_2_-AR-mediated responses (Starke, 2001).

Previous *in vitro* studies showed that vasodilation due to α_2_-AR activation resulted from endothelial NO release. NO-synthase inhibition not only abolished pulmonary vasodilator effects of α_2_-AR agonists but also unmasked their pulmonary vasoconstrictive function in rabbit pulmonary arteries where norepinephrine induced contractions were augmented through α_2_-AR-mediated mechanisms (MacLean et al., 1993). As α_2_-AR agonists may induce pulmonary arterial myocyte contraction (Pepke-Zaba et al., 1993) in addition to relaxation, it is possible that pulmonary vascular responses to α_2_-AR result from the balance between the activation of α_1_- and α_2_-AR-induced smooth muscle cell contraction and endothelial α_2_-AR-mediated vasodilation (Magnenant et al., 2003). Though further experiments will be required to directly address the α_2_-AR-mediated dilation we report in the sheep fetal vessels, we do not believe that this is due to incomplete disruption of the endothelium because bradykinin and acetylcholine have little or no effect on pulmonary arterial reactivity (Blum-Johnston et al., 2016; Giang et al., 2016). Rather, preferential α_2_-AR reactivity in the normoxic fetus and not in other groups suggests that the role for this receptor is labile and greatly influenced by LTH in the fetal period and by postnatal development.

The simplest explanation for α_2_-AR-induced contractile effects is that stimulation of smooth muscle α_2_-ARs inhibits cAMP production *via* G_i_ coupling resulting in increased arterial reactivity. Indeed, there is precedent for direct α_2_-AR-dependent contraction in porcine pulmonary arteries where α_2_c-ARs mediate myocyte contraction when the cytosolic Ca^2+^ concentration is elevated. In these arteries, norepinephrine activates α_2_c-AR following α_1_-AR-stimulation and concomitant myocyte Ca^2+^ increases (Jantschak and Pertz, 2012). Such findings are not restricted to the pulmonary vasculature. In nasal mucosa blood vessels of pigs, which are richly innervated by sympathetic nerves, neurogenic vasomotor contractility is largely regulated through postjunctional α_2_-ARs (Corboz et al., 2013). The relaxation of serotonin preconstricted arteries by α_2_-AR activation may therefore be explained by varied expression of α_2_-AR subtypes or by postjunctional mechanisms. For example, in the pregnant rat myometrium, α_2A_- and α_2C_-AR activation causes myometrial relaxation, while α_2B_-AR stimulation elicits contraction. Thus, it is quite possible there are multiple α_2_-AR subtypes expressed in the pulmonary vasculature that serve distinct roles in regulating arterial relaxation and contraction, which are modified differentially by LTH in the fetal and adult periods (Gaspar et al., 2007). Overall, the current experiments support previous studies suggesting that α_2_-ARs may modulate pulmonary vascular tone and that LTH influences receptor function in sheep vessels (Magnenant et al., 2003; Schindler et al., 2004). Further investigation is required to fully delineate the importance of α_2_-ARs and the roles of prejunctional receptors on perivascular nerves as well as postjunctional receptors on smooth muscle or endothelium.

The present study evaluating α-adrenergic stimulation of the pulmonary vasculature parallels recent studies from our group focused on the cerebral vasculature that compared the effects of maturation and LTH in the fetal and adult periods (Goyal et al., 2010b, 2014). The present study delineates that all three α_1_-AR subtypes are important to normoxic fetal sheep pulmonary arterial reactivity. This compares with normoxic fetal sheep cerebral arteries in which only the α_1B_ and α_1D_-AR regulate arterial contraction (Goyal et al., 2010b). The importance of α_1_-AR subtypes in sheep adult cerebral vessels is also distinct from adult pulmonary vessels. Adult normoxic cerebral vessels are dependent on all three receptor subtypes, while LTH causes pronounced loss in α_1D_-AR function. Adult normoxic pulmonary arteries in comparison are reliant on α_1A_ and α_1B_-AR but not α_1D_-AR activity, and their relative roles are not greatly influenced by LTH. Overall, LTH appears to cause cerebral vessels to become more pulmonary-like in regards to the importance of α_1_-adrenergic receptor subtypes to arterial reactivity (Goyal et al., 2014).

### Alpha-Adrenergic Receptor and Calcium Responses in Pulmonary Arterial Myocytes

Phenylephrine-induced Ca^2+^ signals in individual myocytes within intact arteries were examined to better understand the cellular mechanisms linking α_1_-AR stimulation to vascular reactivity. LTH in the fetal and adult periods caused complex changes in the Ca^2+^ signals. The modest phenylephrine Ca^2+^ activity responses in myocytes of pulmonary arteries from the fetal and adult periods together with the decrease in arterial reactivity relative to high K^+^ in the adult period suggest that arterial contractions in the fetal period are more sensitized to changes in the cytosolic Ca^2+^ concentration. Further, given the increase in arterial reactivity but lack of change in Ca^2+^ activity of arterial myocytes LTH may enhance the Ca^2+^ sensitivity in arterial myocytes from the adult period. Although not examined in the current study, previous work from our group illustrates that there are several intracellular signaling pathways that may be important to changes in the Ca^2+^ sensitivity. Previously, we have shown that rho-kinase is critical to serotonergic and depolarization-induced reactivity in sheep pulmonary arteries (Papamatheakis et al., 2012; Blood et al., 2013). Protein kinase C (PKC) and extracellular regulated kinase (ERK) pathways are also potentially involved as they are controlled by adrenergic receptor activity in cerebral and uterine arteries of sheep (Xiao et al., 2010; Goyal et al., 2010b). In addition, in cerebral arteries from sheep, the different adrenergic receptor isoforms make varied linkages to Ca^2+^, PKC, and ERK pathways (Longo et al., 1996; Ueno et al., 1997; Goyal et al., 2010a,b), which can be modified in unique ways by LTH in the fetal and adult vessels. Elucidating the nuances associated with the changes due to LTH in the fetal and adult periods for the Ca^2+^ sensitization pathways was not addressed in these initial series of studies. However, based on our previous comparisons to vessels from other vascular beds in sheep, we anticipate the mechanistic modifications due to postnatal maturity and LTH to be distinct in the pulmonary vasculature.

### Perspective

The molecular and signaling underpinnings of pulmonary vascular disease are complex. The evidence provided supports the vital role of LTH in the development of adrenergic-induced pulmonary hypertension and the variation in responsiveness due to development. Further, the data substantiate early studies suggesting that adrenergic receptor antagonists can be used to manage the disease (Oriowo et al., 2003) in addition to recent porcine and canine studies (Rothman et al., 2015; Zhou et al., 2015) and a human clinical trial (Chen et al., 2015) focusing on histological, hemodynamic, functional, and clinical benefits to denervation of the pulmonary vasculature. The long-term benefits of the present findings relate to the therapeutic potential of targeting adrenergic signaling in ways that curtail detriments in pulmonary vascular function due to high altitude or other mechanisms that enhance sympathetic drive.

## Data Availability

The datasets generated for this study are available on request to the corresponding author.

## Ethics Statement

This study was carried out in accordance with the recommendations of the regulations of the Animal Welfare Act, the National Institutes of Health Guide for the Care and Use of Laboratory Animals, “The Guiding Principles in the Care and Use of Animals” approved by the Council of the American Physiological Society. The protocol was approved by the Animal Care and Use Committee of Loma Linda University (LLU).

## Author Contributions

SW, LL, DP, LZ, and DPM conceived and designed the research. DP, DPM, QB, SM, MoR, and SV performed the experiments. DP, DPM, DM, QB, SM, MaR, MoR, PG, SV, and SL analyzed data. DM, DP, DPM, QB, SM, MaR, MoR, and SL prepared figures. DM, DP, DPM, PG, LL, LZ, and SW drafted the manuscript. All authors interpreted results of experiments, and with the exception of LL, edited, revised, and approved the final version of manuscript.

### Conflict of Interest Statement

The authors declare that the research was conducted in the absence of any commercial or financial relationships that could be construed as a potential conflict of interest.

## References

[ref1] Allison B. J. Brain K. L. Niu Y. Kane A. D. Herrera E. A. Thakor A. S.. (2016). Fetal *in vivo* continuous cardiovascular function during chronic hypoxia. J. Physiol. 594, 1247–1264. 10.1113/JP271091, PMID: 26926316 PMC4771786

[ref2] Anderson E. A. Sinkey C. A. Mark A. L. (1991). Mental stress increases sympathetic nerve activity during sustained baroreceptor stimulation in humans. Hypertension 17, III43–III49. 10.1161/01.hyp.17.4_suppl.iii43, PMID: 2013492

[ref3] Aranda P. S. Lajoie D. M. Jorcyk C. L. (2012). Bleach gel: a simple agarose gel for analyzing RNA quality. Electrophoresis 33, 366–369. 10.1002/elps.201100335, PMID: 22222980 PMC3699176

[ref4] Barnes P. J. Liu S. F. (1995). Regulation of pulmonary vascular tone. Pharmacol. Rev. 47, 87–131. PMID: 7784481

[ref5] Blood A. B. Terry M. H. Merritt T. A. Papamatheakis D. G. Blood Q. Ross J. M.. (2013). Effect of chronic perinatal hypoxia on the role of rho-kinase in pulmonary artery contraction in newborn lambs. Am. J. Physiol. Regul. Integr. Comp. Physiol. 304, R136–R146. 10.1152/ajpregu.00126.2012, PMID: 23152110 PMC3543659

[ref6] Blum-Johnston C. Thorpe R. B. Wee C. Romero M. Brunelle A. Blood Q.. (2016). Developmental acceleration of bradykinin-dependent relaxation by prenatal chronic hypoxia impedes normal development after birth. Am. J. Physiol. Lung Cell. Mol. Physiol. 310, L271–L286. 10.1152/ajplung.00340.2015, PMID: 26637638 PMC4971892

[ref7] Castillo-Badillo J. A. Sanchez-Reyes O. B. Alfonzo-Mendez M. A. Romero-Avila M. T. Reyes-Cruz G. Garcia-Sainz J. A. (2015). Alpha1B-adrenergic receptors differentially associate with Rab proteins during homologous and heterologous desensitization. PLoS One 10:e0121165. 10.1371/journal.pone.0121165, PMID: 25799564 PMC4370394

[ref8] Chen S. L. Zhang H. Xie D. J. Zhang J. Zhou L. Rothman A. M.. (2015). Hemodynamic, functional, and clinical responses to pulmonary artery denervation in patients with pulmonary arterial hypertension of different causes: phase II results from the pulmonary artery denervation-1 study. Circ. Cardiovasc. Interv. 8:e002837. 10.1161/CIRCINTERVENTIONS.115.002837, PMID: 26553699 PMC4648183

[ref9] Corboz M. R. Rivelli M. A. Shah H. Boyce C. W. Mccormick K. D. Chapman R. W.. (2013). Role of alpha2-adrenoceptors in electrical field stimulation-induced contraction of pig nasal mucosa and pharmacologic characterization of a novel alpha2C-adrenoceptor agonist. Am. J. Rhinol. Allergy 27, 84–90. 10.2500/ajra.2013.27.3842, PMID: 23562194

[ref10] Crossley D. A. 2nd Burggren W. W. Altimiras J. (2003). Cardiovascular regulation during hypoxia in embryos of the domestic chicken *Gallus gallus*. Am. J. Physiol. Regul. Integr. Comp. Physiol. 284, R219–R226. 10.1152/ajpregu.00654.2001, PMID: 12388452

[ref11] Daly I. D. B. Hebb C. (1966). Pulmonary and bronchial vascular systems: Their reactions under controlled conditions of ventilation and circulation. London: Edward Arnold.

[ref12] Ducsay C. A. Goyal R. Pearce W. J. Wilson S. Hu X. Q. Zhang L. (2018). Gestational hypoxia and developmental plasticity. Physiol. Rev. 98, 1241–1334. 10.1152/physrev.00043.2017, PMID: 29717932 PMC6088145

[ref13] Faber J. E. Szymeczek C. L. Cotecchia S. Thomas S. A. Tanoue A. Tsujimoto G.. (2007). Alpha1-adrenoceptor-dependent vascular hypertrophy and remodeling in murine hypoxic pulmonary hypertension. Am. J. Physiol. Heart Circ. Physiol. 292, H2316–H2323. 10.1152/ajpheart.00792.2006, PMID: 17220188

[ref14] Faber J. E. Szymeczek C. L. Salvi S. S. Zhang H. (2006). Enhanced alpha1-adrenergic trophic activity in pulmonary artery of hypoxic pulmonary hypertensive rats. Am. J. Physiol. Heart Circ. Physiol. 291, H2272–H2281. 10.1152/ajpheart.00404.2006, PMID: 16798826

[ref15] Francis M. Qian X. Charbel C. Ledoux J. Parker J. C. Taylor M. S. (2012). Automated region of interest analysis of dynamic Ca(2)+ signals in image sequences. Am. J. Physiol. Cell Physiol. 303, C236–C243. 10.1152/ajpcell.00016.2012, PMID: 22538238 PMC3423022

[ref16] Francis M. Waldrup J. Qian X. Taylor M. S. (2014). Automated analysis of dynamic Ca^2+^ signals in image sequences. J. Vis. Exp. e51560. 10.3791/51560, PMID: 24962784 PMC4195352

[ref17] Garcia-Sainz J. A. Vazquez-Cuevas F. G. Romero-Avila M. T. (2001). Phosphorylation and desensitization of alpha1d-adrenergic receptors. Biochem. J. 353, 603–610. 10.1042/bj3530603, PMID: 11171057 PMC1221606

[ref18] Gaspar R. Gal A. Galik M. Ducza E. Minorics R. Kolarovszki-Sipiczki Z. (2007). Different roles of alpha2-adrenoceptor subtypes in non-pregnant and late-pregnant uterine contractility *in vitro* in the rat. Neurochem. Int. 51, 311–318. 10.1016/j.neuint.2007.06.029 17664026

[ref19] Giang M. Papamatheakis D. G. Nguyen D. Paez R. Blum Johnston C. Kim J.. (2016). Muscarinic receptor activation affects pulmonary artery contractility in sheep: the impact of maturation and chronic hypoxia on endothelium-dependent and endothelium-independent function. High Alt. Med. Biol. 17, 122–132. 10.1089/ham.2015.0116, PMID: 27281473 PMC4913491

[ref20] Gornemann T. Villalon C. M. Centurion D. Pertz H. H. (2009). Phenylephrine contracts porcine pulmonary veins *via* alpha(1B)-, alpha(1D)-, and alpha(2)-adrenoceptors. Eur. J. Pharmacol. 613, 86–92. 10.1016/j.ejphar.2009.04.011, PMID: 19376108

[ref21] Gornemann T. Von Wenckstern H. Kleuser B. Villalon C. M. Centurion D. Jahnichen S.. (2007). Characterization of the postjunctional alpha 2C-adrenoceptor mediating vasoconstriction to UK14304 in porcine pulmonary veins. Br. J. Pharmacol. 151, 186–194. 10.1038/sj.bjp.0707221, PMID: 17375080 PMC2013950

[ref22] Goyal R. Creel K. D. Chavis E. Smith G. D. Longo L. D. Wilson S. M. (2008). Maturation of intracellular calcium homeostasis in sheep pulmonary arterial smooth muscle cells. Am. J. Physiol. Lung Cell. Mol. Physiol. 295, L905–L914. 10.1152/ajplung.00053.2008, PMID: 18776056 PMC2584881

[ref23] Goyal R. Goyal D. Chu N. Van Wickle J. Longo L. D. (2014). Cerebral artery alpha-1 AR subtypes: high altitude long-term acclimatization responses. PLoS One 9:e112784. 10.1371/journal.pone.0112784, PMID: 25393740 PMC4231100

[ref24] Goyal R. Mittal A. Chu N. Arthur R. A. Zhang L. Longo L. D. (2010a). Maturation and long-term hypoxia-induced acclimatization responses in PKC-mediated signaling pathways in ovine cerebral arterial contractility. Am. J. Physiol. Regul. Integr. Comp. Physiol. 299, R1377–R1386. 10.1152/ajpregu.00344.2010 20702800 PMC2980463

[ref25] Goyal R. Mittal A. Chu N. Zhang L. Longo L. D. (2010b). Alpha(1)-adrenergic receptor subtype function in fetal and adult cerebral arteries. Am. J. Physiol. Heart Circ. Physiol. 298, H1797–H1806. 10.1152/ajpheart.00112.2010 20348219 PMC2886655

[ref26] Goyal R. Papamatheakis D. G. Loftin M. Vrancken K. Dawson A. S. Osman N. J.. (2011). Long-term maternal hypoxia: the role of extracellular Ca^2+^ entry during serotonin-mediated contractility in fetal ovine pulmonary arteries. Reprod. Sci. 18, 948–962. 10.1177/1933719111401660, PMID: 21960509 PMC3343111

[ref28] Graven K. K. Farber H. W. (1995). Hypoxia-associated proteins. New Horiz. 3, 208–218. PMID: 7583162

[ref29] Graven K. K. Troxler R. F. Kornfeld H. Panchenko M. V. Farber H. W. (1994). Regulation of endothelial cell glyceraldehyde-3-phosphate dehydrogenase expression by hypoxia. J. Biol. Chem. 269, 24446–24453. PMID: 7929107

[ref30] Hadley S. R. Blood Q. Rubalcava M. Waskel E. Lumbard B. Le P.. (2012). Maternal high-altitude hypoxia and suppression of ryanodine receptor-mediated Ca^2+^ sparks in fetal sheep pulmonary arterial myocytes. Am. J. Physiol. Lung Cell. Mol. Physiol. 303, L799–L813. 10.1152/ajplung.00009.2012, PMID: 22962012 PMC3517681

[ref31] Hawrylyshyn K. A. Michelotti G. A. Coge F. Guenin S. P. Schwinn D. A. (2004). Update on human alpha1-adrenoceptor subtype signaling and genomic organization. Trends Pharmacol. Sci. 25, 449–455. 10.1016/j.tips.2004.06.011, PMID: 15559245

[ref32] Herrera E. A. Pulgar V. M. Riquelme R. A. Sanhueza E. M. Reyes R. V. Ebensperger G.. (2007). High-altitude chronic hypoxia during gestation and after birth modifies cardiovascular responses in newborn sheep. Am. J. Physiol. Regul. Integr. Comp. Physiol. 292, R2234–R2240. 10.1152/ajpregu.00909.2006, PMID: 17322112

[ref33] Herrera E. A. Riquelme R. A. Ebensperger G. Reyes R. V. Ulloa C. E. Cabello G.. (2010). Long-term exposure to high-altitude chronic hypoxia during gestation induces neonatal pulmonary hypertension at sea level. Am. J. Physiol. Regul. Integr. Comp. Physiol. 299, R1676–R1684. 10.1152/ajpregu.00123.2010, PMID: 20881096 PMC3007194

[ref34] Huang J. Xie T. Wu Y. Li X. Lusina S. Ji E. S.. (2010). Cyclic intermittent hypoxia enhances renal sympathetic response to ICV ET-1 in conscious rats. Respir. Physiol. Neurobiol. 171, 83–89. 10.1016/j.resp.2010.03.008, PMID: 20227529 PMC2884044

[ref35] Hyman A. L. Kadowitz P. J. (1989). Analysis of responses to sympathetic nerve stimulation in the feline pulmonary vascular bed. J. Appl. Physiol. 67, 371–376. 10.1152/jappl.1989.67.1.371, PMID: 2569455

[ref36] Jantschak F. Pertz H. H. (2012). Alpha2C-adrenoceptors play a prominent role in sympathetic constriction of porcine pulmonary arteries. Naunyn Schmiedeberg's Arch. Pharmacol. 385, 595–603. 10.1007/s00210-012-0741-3 22371269

[ref37] Kamitomo M. Alonso J. G. Okai T. Longo L. D. Gilbert R. D. (1993). Effects of long-term, high-altitude hypoxemia on ovine fetal cardiac output and blood flow distribution. Am. J. Obstet. Gynecol. 169, 701–707. 8372883 10.1016/0002-9378(93)90646-z

[ref38] Kamitomo M. Longo L. D. Gilbert R. D. (1994). Cardiac function in fetal sheep during two weeks of hypoxemia. Am. J. Phys. 266, R1778–R1785. 10.1152/ajpregu.1994.266.6.R1778, PMID: 8024028

[ref39] Levy M. Tulloh R. M. Komai H. Stuart-Smith K. Haworth S. G. (1995). Maturation of the contractile response and its endothelial modulation in newborn porcine intrapulmonary arteries. Pediatr. Res. 38, 25–29. 10.1203/00006450-199507000-00005, PMID: 7478792

[ref40] Llanos A. J. Ebensperger G. Herrera E. A. Reyes R. V. Pulgar V. M. Seron-Ferre M. (2011). Fetal and postnatal pulmonary circulation in the alto Andino. Placenta 32(Suppl. 2), S100–S103. 10.1016/j.placenta.2011.01.001 21295346

[ref41] Longo L. D. Ueno N. Zhao Y. Zhang L. Pearce W. J. (1996). NE-induced contraction, alpha 1-adrenergic receptors, and ins(1,4,5)P3 responses in cerebral arteries. Am. J. Phys. 270, H915–H923. 10.1152/ajpheart.1996.270.3.H915, PMID: 8780186

[ref42] MacLean M. R. McCulloch K. M. (1998). Influence of applied tension and nitric oxide on responses to endothelins in rat pulmonary resistance arteries: effect of chronic hypoxia. Br. J. Pharmacol. 123, 991–999. 10.1038/sj.bjp.0701682, PMID: 9535030 PMC1565238

[ref43] MacLean M. R. McCulloch K. M. McGrath J. C. (1993). Influences of the endothelium and hypoxia on alpha 1- and alpha 2-adrenoceptor-mediated responses in the rabbit isolated pulmonary artery. Br. J. Pharmacol. 108, 155–161. 10.1111/j.1476-5381.1993.tb13456.x, PMID: 8094023 PMC1907688

[ref44] Magnenant E. Jaillard S. Deruelle P. Houfflin-Debarge V. Riou Y. Klosowski S.. (2003). Role of the alpha2-adrenoceptors on the pulmonary circulation in the ovine fetus. Pediatr. Res. 54, 44–51. 10.1203/01.PDR.0000065726.43910.91, PMID: 12646721

[ref45] Mandel J. Taichman D. (2006). Pulmonary vascular disease. Philadelphia, PA: Saunders Elsevier.

[ref46] McGillick E. V. Orgeig S. Allison B. J. Brain K. L. Niu Y. Itani N.. (2017). Maternal chronic hypoxia increases expression of genes regulating lung liquid movement and surfactant maturation in male fetuses in late gestation. J. Physiol. 595, 4329–4350. 10.1113/JP273842, PMID: 28318025 PMC5491863

[ref47] Michel M. C. Wieland T. Tsujimoto G. (2009). How reliable are G-protein-coupled receptor antibodies? Naunyn Schmiedeberg's Arch. Pharmacol. 379, 385–388. 10.1007/s00210-009-0395-y, PMID: 19172248

[ref48] Niermeyer S. (2007). Going to high altitude with a newborn infant. High Alt. Med. Biol. 8, 117–123. 10.1089/ham.2007.1068, PMID: 17584005

[ref49] Ogata M. Ohe M. Katayose D. Takishima T. (1992). Modulatory role of EDRF in hypoxic contraction of isolated porcine pulmonary arteries. Am. J. Phys. 262, H691–H697. 10.1152/ajpheart.1992.262.3.H691, PMID: 1558177

[ref50] Ok S. H. Kwon S. C. Baik J. Hong J. M. Oh J. Han J. Y.. (2016). Dexmedetomidine-induced contraction involves CPI-17 phosphorylation in isolated rat aortas. Int. J. Mol. Sci. 17:1663. 10.3390/ijms17101663, PMID: 27706026 PMC5085696

[ref51] Oriowo M. A. Chandrasekhar B. Kadavil E. A. (2003). Alpha 1-adrenoceptor subtypes mediating noradrenaline-induced contraction of pulmonary artery from pulmonary hypertensive rats. Eur. J. Pharmacol. 482, 255–263. 10.1016/j.ejphar.2003.10.001 14660030

[ref52] Papamatheakis D. G. Blood A. B. Kim J. H. Wilson S. M. (2013). Antenatal hypoxia and pulmonary vascular function and remodeling. Curr. Vasc. Pharmacol. 11, 616–640. 10.2174/1570161111311050006, PMID: 24063380 PMC4527655

[ref53] Papamatheakis D. G. Patel J. J. Blood Q. Merritt T. T. Longo L. D. Wilson S. M. (2012). Depolarization-dependent contraction increase after birth and preservation following long-term hypoxia in sheep pulmonary arteries. Pulm. Circ. 2, 41–53. 10.4103/2045-8932.94832, PMID: 22558519 PMC3342748

[ref54] Papamatheakis D. G. Vemulakonda S. Blood Q. Goyal R. Rubalcava M. Vrancken K.. (2011). Preservation of serotonin-mediated contractility in adult sheep pulmonary arteries following long-term high-altitude hypoxia. High Alt. Med. Biol. 12, 253–264. 10.1089/ham.2010.1076, PMID: 21962069 PMC3186692

[ref55] Patel K. P. Li Y. F. Hirooka Y. (2001). Role of nitric oxide in central sympathetic outflow. Exp. Biol. Med. 226, 814–824. 10.1177/153537020122600902 11568303

[ref56] Peng Y. J. Overholt J. L. Kline D. Kumar G. K. Prabhakar N. R. (2003). Induction of sensory long-term facilitation in the carotid body by intermittent hypoxia: implications for recurrent apneas. Proc. Natl. Acad. Sci. USA 100, 10073–10078. 10.1073/pnas.1734109100 12907705 PMC187770

[ref57] Pepke-Zaba J. Higenbottam T. W. Dinh-Xuan A. T. Ridden C. Kealey T. (1993). Alpha-adrenoceptor stimulation of porcine pulmonary arteries. Eur. J. Pharmacol. 235, 169–175. 10.1016/0014-2999(93)90133-3, PMID: 8389712

[ref58] Rothman A. M. Arnold N. D. Chang W. Watson O. Swift A. J. Condliffe R.. (2015). Pulmonary artery denervation reduces pulmonary artery pressure and induces histological changes in an acute porcine model of pulmonary hypertension. Circ. Cardiovasc. Interv. 8:e002569. 10.1161/CIRCINTERVENTIONS.115.002569, PMID: 26553697 PMC4648184

[ref59] Salvi S. S. (1999). Alpha1-adrenergic hypothesis for pulmonary hypertension. Chest 115, 1708–1719. 10.1378/chest.115.6.1708, PMID: 10378571

[ref60] Schindler M. B. Hislop A. A. Haworth S. G. (2004). Postnatal changes in response to norepinephrine in the normal and pulmonary hypertensive lung. Am. J. Respir. Crit. Care Med. 170, 641–646. 10.1164/rccm.200311-1551OC, PMID: 15184201

[ref61] Schonbrunn A. (2014). Editorial: antibody can get it right: confronting problems of antibody specificity and irreproducibility. Mol. Endocrinol. 28, 1403–1407. 10.1210/me.2014-1230, PMID: 25184858 PMC4154242

[ref62] Shebuski R. J. Ohlstein E. H. Smith J. M. Jr. Ruffolo R. R. Jr. (1987). Enhanced pulmonary alpha-2 adrenoceptor responsiveness under conditions of elevated pulmonary vascular tone. J. Pharmacol. Exp. Ther. 242, 158–165. PMID: 3039109

[ref63] Shen C. P. Romero M. Brunelle A. Wolfe C. Dobyns A. Francis M.. (2018). Long-term high-altitude hypoxia influences pulmonary arterial L-type calcium channel-mediated Ca(2+) signals and contraction in fetal and adult sheep. Am. J. Physiol. Regul. Integr. Comp. Physiol. 314, R433–R446. 10.1152/ajpregu.00154.2017, PMID: 29167165 PMC5899247

[ref64] Starke K. (2001). Presynaptic autoreceptors in the third decade: focus on alpha2-adrenoceptors. J. Neurochem. 78, 685–693. 10.1046/j.1471-4159.2001.00484.x, PMID: 11520889

[ref65] Thiriet M. (2013). Anatomy and physiology of the circulatory and ventilatory systems. New York: Springer.

[ref66] Toyama H. Wagatsuma T. Ejima Y. Matsubara M. Kurosawa S. (2009). Cesarean section and primary pulmonary hypertension: the role of intravenous dexmedetomidine. Int. J. Obstet. Anesth. 18, 262–267. 10.1016/j.ijoa.2008.08.001, PMID: 19157850

[ref67] Tucker A. Mcmurtry I. F. Reeves J. T. Alexander A. F. Will D. H. Grover R. F. (1975). Lung vascular smooth muscle as a determinant of pulmonary hypertension at high altitude. Am. J. Phys. 228, 762–767. 10.1152/ajplegacy.1975.228.3.762 234690

[ref68] Ueno N. Zhao Y. Zhang L. Longo L. D. (1997). High altitude-induced changes in alpha1-adrenergic receptors and ins(1,4,5)P3 responses in cerebral arteries. Am. J. Phys. 272, R669–R674. 10.1152/ajpregu.1997.272.2.R669, PMID: 9124493

[ref69] Villamor E. Kessels C. G. Ruijtenbeek K. Van Suylen R. J. Belik J. De Mey J. G.. (2004). Chronic in ovo hypoxia decreases pulmonary arterial contractile reactivity and induces biventricular cardiac enlargement in the chicken embryo. Am. J. Physiol. Regul. Integr. Comp. Physiol. 287, R642–R651. 10.1152/ajpregu.00611.2003, PMID: 15117730

[ref70] Weir E. K. Archer S. L. Reeves J. T. (2000). The fetal and neonatal pulmonary circulations. Armonk, NY: Futura Pub.

[ref71] Xiao D. Huang X. Longo L. D. Zhang L. (2010). PKC regulates alpha(1)-adrenoceptor-mediated contractions and baseline Ca(2+) sensitivity in the uterine arteries of nonpregnant and pregnant sheep acclimatized to high altitude hypoxia. High Alt. Med. Biol. 11, 153–161. 10.1089/ham.2009.1076, PMID: 20586600 PMC3114155

[ref72] Xiao D. Huang X. Pearce W. J. Longo L. D. Zhang L. (2003). Effect of cortisol on norepinephrine-mediated contractions in ovine uterine arteries. Am. J. Physiol. Heart Circ. Physiol. 284, H1142–H1151. 10.1152/ajpheart.00834.2002, PMID: 12531736

[ref73] Xue Q. Ducsay C. A. Longo L. D. Zhang L. (2008). Effect of long-term high-altitude hypoxia on fetal pulmonary vascular contractility. J. Appl. Physiol. 104, 1786–1792. 10.1152/japplphysiol.01314.2007, PMID: 18388246

[ref74] Zhou L. Zhang J. Jiang X. M. Xie D. J. Wang J. S. Li L.. (2015). Pulmonary artery denervation attenuates pulmonary arterial remodeling in dogs with pulmonary arterial hypertension induced by dehydrogenized monocrotaline. JACC Cardiovasc. Interv. 8, 2013–2023. 10.1016/j.jcin.2015.09.015, PMID: 26738673

[ref75] Zoccal D. B. Simms A. E. Bonagamba L. G. Braga V. A. Pickering A. E. Paton J. F.. (2008). Increased sympathetic outflow in juvenile rats submitted to chronic intermittent hypoxia correlates with enhanced expiratory activity. J. Physiol. 586, 3253–3265. 10.1113/jphysiol.2008.154187, PMID: 18450774 PMC2538770

